# How would patients with psychosis like to be in contact with a volunteer: Face-to-face or digitally?

**DOI:** 10.1371/journal.pone.0216929

**Published:** 2019-05-16

**Authors:** Mariana Pinto da Costa, Agnes Chevalier, Aida Farreny, Megan Cassidy, Monica Leverton, Sarah Toner, Stefan Priebe

**Affiliations:** 1 Unit for Social and Community Psychiatry (WHO Collaborating Centre for Mental Health Services Development), Queen Mary University of London, London, United Kingdom; 2 Hospital de Magalhães Lemos, Porto, Portugal; 3 Institute of Biomedical Sciences Abel Salazar (ICBAS), University of Porto, Porto, Portugal; Maastricht Universitair Medisch Centrum+, NETHERLANDS

## Abstract

**Introduction:**

Volunteer befriending can be used to address social isolation in patients with psychosis. Traditionally this involves face-to-face encounters between a volunteer and a patient, but modern digital technology also makes it possible to have these interactions remotely. This study aimed to explore the views and interests of patients with psychosis about different formats of volunteering, face-to-face or digitally.

**Methods:**

A survey was conducted with patients with psychotic disorders in community mental health teams in London. Questions covered socio-demographic characteristics, quality of life, loneliness, views on the different formats of volunteering and types of volunteers, and their interest in getting volunteering support, face-to-face or digitally. Binary logistic regressions were used to investigate potential predictors of interest in getting volunteering support face-to-face or digitally.

**Results:**

A total of 151 patients with psychotic disorders were included in this study. More than half of the patients (n = 87, 57.6%) had not heard about these volunteering programs. Many were interested in getting face-to-face (n = 87, 57.6%) and digital (n = 56, 37.1%) volunteering. For the face-to-face encounters, most preferred them to be weekly (n = 36, 41.4%), for one-hour (n = 32, 36.8%), and with an open-ended relationship (n = 45, 51.7%). For the digital contacts, most preferred them to be weekly (n = 17, 30.9%) and through text messages (n = 26, 46.4%). A minority of patients (n = 20, 13.2%) did not use digital technology. Patients with lower quality of life were significantly more likely to prefer face-to-face volunteering (p < .05). Younger patients and with fewer years of diagnosis were significantly more likely to prefer digital volunteering (p < .05).

**Conclusions:**

The variability in patients’ interests suggests that different formats of volunteer support should be offered. Digital volunteering may become more important in the future, since many younger patients are interested in it.

## Introduction

Psychosis comprises a group of severe psychiatric disorders in which a person’s perceptions, thoughts, mood and behaviour are significantly altered [1]. In the currently most used diagnostic classification systems of psychiatric disorders, such as the International Classification of Diseases-10 (ICD-10) [2] and the Diagnostic and Statistical Manual-5 (DSM5) ] [3], psychosis is narrowly defined by the presence of hallucinations (without insight of their pathologic nature), delusions or both.

Whilst the evidence linking environmental risks and psychosis incidence has been recognised [4,5], psychosis is still a contributor to disability and a barrier to productivity and participation [6,7]. For these reasons, psychosocial interventions should be part of the treatment plan for people with psychosis, especially during the stable phase of their illness [8].

People with psychotic disorders experience difficulties in establishing and maintaining social relationships, have less social support [9], and experience higher levels of social isolation and loneliness than the general population [10,11]. These difficulties are not only linked with their symptoms and consequences of their disorder, such as decreased social functioning, withdrawal and deficits in communication [12], but also with stigma and discrimination towards them [13]. Importantly, patients’ social isolation is linked with poor illness outcomes [14], both in mental and physical health [15,16]. In fact, although many patients would like to have more friends [17], few interventions exist to address social isolation in patients with psychosis [18,19].

In the United Kingdom 14.2 million people formally volunteer at least once a month [20], representing a vital resource for communities [21]. Volunteer befriending can be a way to promote social relationships [22,23] and positive attitudes towards people with psychotic disorders [24], and previous research has showed its value [25]. Such volunteering programs can have different structures and purposes, recruiting different types of volunteers and encouraging different types of relationships to be formed [26]. These differences may play a role in the type of relationship established (in the friendship/professional therapeutic spectrum), in the benefit patients may have from the volunteers input (being in contact with a befriender, who is a general person from the community, or a peer, who is someone who shares the experience of having received mental health care) and the impact of the relationship for different volunteers (e.g. changing the attitudes towards people with mental health disorders in befrienders, or contributing to the own recovery of those providing peer-support).

Usually these volunteer-patient interactions take place in person. However, some communities (and countries) may face barriers to these face-to-face encounters. Logistical problems such as long travel commutes, busy schedules, other commitments of volunteers, or patients’ difficulties leaving the house, may all hinder face-to-face encounters [27]. For some patients, remote contacts through new digital technology might be an acceptable and appealing method, which would substantially widen the options for volunteering, either as a complement or a replacement to the face-to-face encounters. Digital technology broadens the ways to interact and communicate with other people (e.g. writing/reading text messages or e-mails, speaking/listening through audio calls or seeing each other in video calls) adding a pool of possible more or less synchronous interactions between patients and volunteers.

Thus, the question arises as to how many and which patients are interested in getting digital volunteering input and what type of contacts they prefer. We addressed this question using the dataset of a survey among patients in community mental health care. Whilst in a previous publication of this study we looked at patients’ general preferences of volunteering [28], in this article we focused on the interests of getting volunteering input face-to-face or digitally. We have also focused on patients with psychotic disorders rather than on a diagnostically mixed sample as in the previous publication [28].

In particular we aimed to identify the extent of patients interests in volunteering in mental health (either face-to-face or using technology), and how socio-demographic and other individual patients characteristics predict their interests.

## Materials and methods

### Study design

A cross-sectional survey was carried out in nine community mental health teams (CMHTs) across East London. A favourable opinion was given by the National Research Ethics Service Committee South West–Exeter of the Health Research Authority (Ref 14/SW/1011) who approved this study. More information about the survey can be retrieved elsewhere [28,29].

### Eligibility criteria

The inclusion criteria were: (i) aged over 18; (ii) receiving secondary mental health care in outpatients services; (iii) diagnosed with a psychotic disorder (F20-F29), according to the International Statistical Classification of Diseases and Related Health Problems 10 [30]; (iv) ability to speak English well enough to understand the consent process and the survey questions. Exclusion criteria were lack of capacity to consent at the time of the interview and already being a participant in a trial of face-to-face volunteer befriending [31].

### Procedure

Eligible participants were identified in nine community mental health teams in East London. Patients were invited to participate on the day of their appointment with a clinician to explain the study’s aims, provide more information if requested and obtain informed written consent prior to assessment. All face-to-face interviews were conducted in English by experienced researchers, between August 2016 and August 2017.

### Instruments

The survey included questions about socio-demographic characteristics, patients’ views on several aspects of volunteer befriending, such as preferences for volunteers’ characteristics (e.g. with or without experience as a patient in mental health care), the format of the interactions (face-to-face or digitally), and the patients’ interest in receiving such input. In addition, the survey also measured quality of life (MANSA) [32], and assessed whether patients have a close friend or have seen a friend in the last week. Loneliness was measured using an item from the WHO Quality of Life Assessment [33].

### Statistical analysis

Descriptive statistics were used to report the percentages for categorical variables and mean values with standard deviations for continuous variables. Missing data were omitted on an analysis-by-analysis basis and valid percentages are reported.

Living arrangements (living alone, with partner, with parents, with children, or others) and employment status (paid employment, in sheltered employment, in training/education, unemployed, retired or other) were recoded as dichotomous variables (“live alone” and “live with others”; “in employment” and “not in employment”). Similarly, digital interest (“yes”, “no”, “do not use technology”) was treated as a dichotomous variable, excluding those that do not use technology (internet, computer or a phone) as missing, to test for predictors of its interest.

Univariable binary logistic regression was used to assess whether patients characteristics were related to the dependent variables, “interest in face-to-face volunteering” and “interest in digital volunteering”. When the significant variables in the univariable analyses (p<0.05) were not strongly correlated with each other, multivariable binary logistic regression models were performed, adjusting for all the other variables.

Data analysis was conducted using the Software Package for Social Sciences for Windows v. 24.0 (SPSS Inc. Chicago, IL).

## Results

In total, 898 patients were screened for inclusion in this study across nine CMHTs in East London NHS Foundation Trust. We approached 699 patients, but 412 refused. Overall, 217 consented to take part in the interviews, of which 66 were excluded (either as they were in an ongoing volunteering research trial, had ineligible diagnosis, insufficient capacity or had already been recruited). Therefore 151 patients were included for this analysis.

### Socio-demographics

This sample of 151 patients (Table 1) was ethnically diverse, being the majority black (41.7%). The majority were male, and the mean age was 42.6 years old (SD = 11.1, range 20–68), with a mean of 15.6 years (SD = 9.7) since receiving their diagnosis. All the patients were followed in a CMHT, and all were taking medication. In regard to employment status, the majority (93.3%) were not employed, with a mean of monthly income of £ 699.69 (SD = 343.0), and all received state benefits. Concerning household arrangements, most lived alone (68.3%), and the majority (59.9%) did not have children. In the quality of life assessment, 63.6% of the patients reported having a close friend and 53.6% saw a friend in the last week. In regard to their current feeling of loneliness, 36.0% expressed not feeling lonely at all, 23.3% slightly, 20.0% moderately, 10.7% very and 10.0% extremely.

**Table 1:** Socio-demographic characteristics.

Socio-demographics	N (%)
**Gender**	
Male	108 (71.5)
Female	43 (28.5)
**Ethnicity**	
White	35 (23.2)
Black Caribbean	22 (14.6)
Black African	29 (19.2)
Black Other	12 (7.9)
Bangladeshi	19 (12.6)
Indian	5 (3.3)
Asian Pakistani	8 (5.3)
Asian Chinese	2 (1.3)
Other	19 (12.6)
**Living arrangements**	
Live alone	99 (68.3)
Live with parents	18 (12.4)
Live with partner	9 (6.2)
Live with children	5 (3.4)
Live with others	14 (9.7)
**Employment**	
In paid employment	9 (6.0)
In sheltered employment	1 (0.7)
Unemployed	126 (84.0)
In training/education	5 (3.3)
Retired	5 (3.3)
Other	4 (2.7)

The preferences of patients with psychosis about different aspects of volunteer befriending varied regarding the frequency, duration and goal of the relationship, the type and role of volunteers, and the responsibility of the organisations. These findings are outlined below, comparing the views of those patients interested in face-to-face encounters with those interested in digital interactions.

### Knowledge and interest in face-to-face and digital volunteering

More than half of the patients (n = 87, 57.6%) had not heard about these volunteering programs previously. Several patients were interested in getting face-to-face (n = 87, 57.6%) and digital (n = 56, 37.1%) volunteering support. Few patients (n = 20, 13.2%) did not use technology (internet, computer, phone).

In the univariable regression, a significant association was found between the interest in getting face-to-face volunteering input with loneliness and quality of life as significant predictors (Table 2). On the other hand, interest in getting digital volunteering input was predicted by age and years since diagnosis (Table 3).

**Table 2:** Univariable and multivariable logistic regression on the interest in getting face-to-face volunteering input.

		Univariable analysis				Multivariable analysis			
	Variables	OR	95% CI		*p*	OR	95% CI		*p*
**Face-to-face** **volunteering**	Age	.996	.967	1.026	.800				
	Gender	.658	.316	1.372	.264				
	Living arrangements	.874	.714	1.069	.191				
	Income	1.000	.999	1.002	.659				
	Years since dx	.945	.965	1.034	.945				
	Hospital last year	.870	.441	1.716	.687				
	Weekly work hours	.983	.929	1.041	.563				
	Time use (hours)	.954	.870	1.045	.310				
	Social contacts	.956	.817	1.120	.579				
	Loneliness	1.383	1.063	1.799	.016	1.182	.879	1.591	.268
	Quality of life	.582	.403	.841	.004	.626	.413	.948	.027

**Table 3:** Univariable logistic regression on the interest in getting volunteering input digitally.

		Univariable analysis			
	Variables	OR	95% CI	95% CI	*p*
**Digital volunteering**	Age	.957	.924	.991	.014
	Gender	.855	.393	1.857	.692
	Living arrangements	1.017	.816	1.268	.878
	Income	.999	.997	1.001	.438
	Years since dx	.956	.915	.999	.045
	Hospital last year	1.424	.687	2.954	.342
	Weekly work hours	1.025	.966	1.088	.408
	Time use (hours)	1.074	.969	1.190	.172
	Social contacts	.994	.841	1.176	.947
	Loneliness	1.089	.837	1.417	.525
	Quality of life	.737	.504	1.078	.116

In the multivariable regression, in regards to the interest in getting face-to-face volunteering input, only quality of life remained significant (p<0.05), with lower quality of life indicating higher interest. We did not conduct multivariable analysis for the interest in getting digital volunteering input, since the only two significant variables found in the univariable analysis were strongly correlated with each other.

### Frequency and format of the contacts

The majority of patients preferred face-to-face weekly encounters (n = 36, 41.4%), followed by: more than once a week (n = 24, 27.6%), monthly (n = 13, 14.9%), fortnightly (n = 11, 12.6%), less than once per month (n = 1, 1.1%) or no preference (n = 2, 2.3%). Most patients favoured encounters of one hour (n = 32, 36.8%), whereas the rest preferred more than two hours (n = 18, 20.7%), half an hour (n = 17, 19.5%), two hours (n = 16, 18.4%), less than half an hour (n = 3, 3.4%) or had no preference (n = 1, 1.1%). In regard to the duration of the relationship, most preferred it to be open-ended (n = 45, 51.7%), followed by six months (n = 14, 16.1%), one year (n = 5, 5.7%), more than one year (n = 4, 4.6%), three months (n = 3, 3.4%), nine months (n = 3, 4.4%), one month (n = 3, 3.4%) or no preference (n = 10, 11.5%).

Preferences for the frequency of digital interactions was most often once per week (n = 17, 30.9%) and every other day (n = 16, 29.1%), followed by every two weeks (n = 6, 10.9%), monthly (n = 5, 9.1%) or less often than once per month (n = 1, 1.8%). The preferred means of contact were via text message (n = 26, 46.4%) and WhatsApp (n = 14, 25.0%), followed by email (n = 6, 10.7%), Skype (n = 5, 8.9%), Facebook or other social network (n = 3, 5.4%) and phone-calls (n = 2, 3.6%).

### Type of volunteer

For both patients interested in getting face-to-face and digital volunteering input, the majority preferred having a volunteer who had lived experience as a patient in mental health care. The rest either did not want someone with experience as a patient or had no preference (Table 4).

**Table 4:** Patient’s preferences for the type of volunteer according to their digital interest.

		Digitally interested	Not digitally interested
**Type of volunteer**	With lived experience as a patient in mental health care	37 (66.1%)	34 (48.6%)
	Without lived experience as a patient in mental health care	8 (14.3%)	22 (31.4%)
	No preference	11(19.6%)	14 (20.0%)

### Aim of the volunteering and type of relationship

When comparing the views of patients interested in getting digital volunteering input with those that are not, preferences for the overall aim of volunteering differed (Table 5).

**Table 5:** Patient’s preferences for the aim of volunteering according to their digital interest.

		Digitally interested	Not digitally interested
**Aim of volunteering**	Make a new friend	32 (57.1%)	28 (41.2%)
	Do more activities	24 (42.9%)	40 (58.8%)

Within those digitally interested, more aimed to make a new friend than to do more activities. Whereas among those not digitally interested, more aimed to do more activities than to make a new friend.

In addition, when comparing the preferred type of relationship between those patients that are digitally interested with those that are not, preferences also varied (Table 6). Those digitally interested mostly wanted a real friendship and those not digitally interested mostly wanted someone who talks and listens to them.

**Table 6:** Patients’ preferred type of relationship according to their digital interest in volunteering.

		Digitally interested	Not digitally interested
**Type of relationship**	A real friendship	21 (38.2%)	14 (20.3%)
	Someone who talks with me and listens	10 (18.2%)	27 (39.1%)
	Someone who does activities with me	16 (29.1%)	10 (14.5%)
	Other preferences	8 (14.5%)	18 (26.1%)

### Role and responsibility of the volunteers and the organisations

For both patients interested in face-to-face and digital volunteering, the majority would like volunteers to be in contact with their mental health team (73.8% and 65.5% respectively), whereas the rest would prefer them not to.

Concerning the costs of activities, for those patients interested in face-to-face volunteering, many patients believed that the organisation should pay for them in full (n = 37, 45.1%), whereas the rest thought costs should be divided between the organisation and the patient (n = 23, 28.0%), or that the patients should cover their own costs fully (n = 22, 26.8%). For those patients interested in digital volunteering, if costs arise, most felt the organisation should contribute to the payment (43.4%, n = 23) or pay it fully (n = 21, 39.6%), with a few considering that patients should pay for these completely (n = 9, 17.0%).

## Discussion

### Main findings

A significant proportion of patients with psychotic disorders expressed interest in face-to-face and remote digital volunteering. The face-to-face format of a one-hour weekly meeting, with an open-ended relationship was preferred. For the digital contacts, most preferred once per week and through text messages.

Both in patients interested in face-to-face and digital volunteering, the majority wanted: i) to have a volunteer with experience of being a patient in mental health care, ii) the volunteer to be in contact with their mental health team, and iii) the organisations to contribute to the costs.

However, differences were found in regard to the aim of the volunteering and the type of relationship preferred between those digitally interested and those not. Patients who were interested in getting in contact with a volunteer digitally mostly wanted a real friendship. Patients interested in face-to-face meetings preferred a volunteer with whom they could talk, and were less interested in a friendship. One of the reasons for this could be that patients interested in digital contacts, see technology as a way to establish contact with other people, and to form friendships.

In the univariable regression, higher loneliness and lower quality of life were significant predictors of interest in getting face-to-face volunteering input, whereas for digital volunteering, being younger and having a more recent diagnosis were found to be significant predictors. In the multivariable prediction model only lower quality of life was a significant predictor of the interest in getting face-to-face volunteering input. These findings suggest that patients’ lower quality of life is a key contributor for patients’ interest in face-to-face volunteering programs, and highlights that younger people and with fewer years of diagnosis are generally more interested in interventions provided digitally.

### Strengths and limitations

This study has been the first to explore and compare the views and interests of patients with psychosis in being in contact with a volunteer face-to-face or digitally. As this sample was recruited from CMHTs and not from volunteering programs, this enabled us to capture variations in patients’ interests for face-to-face and digital interactions, regardless of their prior experiences with volunteering. Further strengths are that patients were personally interviewed by trained researchers rather than responding to a postal questionnaire and that the sample was diagnostically homogeneous.

Despite its originality, the study has several limitations. The sample is selective and it is difficult to estimate how the percentages found in this study translate into figures of larger and more representative patient samples. Whilst this may have influenced the results regarding patient preferences, associations are usually more robust against selection bias, and the predictor analysis should be less affected by the potential selection bias. The sample was recruited in East London, a very multi-ethnic and traditionally deprived inner city area. As such, patients’ views may differ from those in other areas in the United Kingdom or in other countries. It therefore may not be appropriate to generalise these findings to the whole city or country.

Despite the focus on patients’ views and interests in taking part in these programs, the actual past experience of patients with volunteering has not been assessed in this study. In fact, although patients were given a brief description of volunteer befriending before completing the survey, it is unclear whether a more comprehensive understanding or personal previous experience on such programs would impact upon expressing or reiterating interest to get volunteer input.

Therefore, regardless of the interest to engage in volunteering, positive responses expressed in the survey may not accurately reflect behaviour. It is unclear how those interested in taking part in face-to-face or digital volunteering would in fact respond if they were offered the opportunity to engage in such initiatives.

Lastly, we have not assessed psychotic experiences and symptoms of the participants. All of the participants were deemed well enough by their clinicians, having capacity to participate in the study. Yet, we are aware that psychosocial interventions are most critical during the stable phase of illness, and not having explored this may be a limitation of this study.

### Comparisons with the literature

Most of the literature on volunteering programs mainly focuses on volunteers’ experiences and organisational descriptions. In fact, volunteering programs depend on human resources, such as staff (that manage them) and the volunteers (that provide their free time to collaborate with them). This reality may explain why up until now, patients’ views and interests in volunteering have not been explored much, possibly because they have not been seen as important to the effective running of such programs. Therefore, little information is available on patients’ views to be compared with the findings from this study.

The sample was mostly composed of male patients, which is in line with the epidemiology of psychotic disorders [34]; yet patients that actually engage with volunteers are usually female [35]. Similarly, the current findings on patients’ quality of life [36], loneliness and social contacts [37] are consistent with previous studies of the same diagnostic group.

These results show an interest of patients in having remote digital volunteering, and adds to what is known about the use of technology by patients with schizophrenia [38–40,41]. Moreover, these results show the preferences between various digital communication methods, where text messages were favoured over other means, such as phone or video calls. A previous study with young adults with a first psychotic episode found that young people preferred a combination of several technologies to receive mental health care (as text, video and audio), and amongst those methods, text messages were also the preferred option [42]. In addition, concerning the duration of the relationship, the majority of patients preferred them to be open-ended, even if most of the organisations require a minimum time commitment from volunteers of one year [22]. These preferences may suggest that patients favour less strict and synchronous methods of communication, having more time to decide when to interact. This could be since in more asynchronous communications people do not wait around for an immediate reply, whereas more real time synchronous communication may pressure people to interact.

These results also suggest that younger patients with fewer years of diagnosis are more interested in digital methods, which resonates with previous associations found with age [43] and cognitive abilities (eg. memory and speed of processing) as predictors of technology usage in the general population [44].

An important finding has been that the majority of the patients would like to have a volunteer with lived experience in mental health, preferring the format of a peer. However, this preference may lead to difficulties while trying to categorise the volunteers, as it requires them to self-disclose whether they have received mental health care at any point in their life, and some may prefer not to report it [45], joining a volunteering program as a regular befriender and not as a self-disclosed peer.

Finally, patients would like for volunteers to be in contact with the mental health teams, which many not be current standard practice. Equally, although it has been recommended that the costs of the activities are subsidised by the volunteering organisations [46], in many occasions these are covered by the volunteers or the patients themselves [47].

### Relevance of the findings and implications for practice, policies and research

These findings represent the interests of patients with psychosis and may be used to inform the development and organisation of current and future face-to-face and digital volunteering programs. This is vital, as patients’ perspectives and input on their care has been increasingly recognised as important in health services [48,49].

It is unclear how specific these results are to the patients in East London, and future studies should explore whether these findings differ for patients in the rest of the country or abroad. Further research should also address the views of volunteers and mental health professionals in East London and compare commonalities and differences across these views.

The fact that nearly half of the patients had not heard about volunteering befriending programs raises awareness that more promotion is required in order to increase the knowledge of these opportunities for patients in the community, providing them with the option to engage if interested. Therefore, these findings may have implications for how social interventions, such as volunteering are promoted and advertised to patients.

The variability in patients’ interests suggests that volunteering should be offered in different formats and with enough flexibility to incorporate individual preferences.

Digital tools are increasingly being considered as a promising avenue for improving access to and quality of mental health care [50]. This is particularly true for young people given the omnipresence of digital technologies in their daily lives. Therefore, it is arguably the right time for technology to support and complement many areas of mental health care, and as such, provide volunteer support. Such digital programs are likely to widen the options for volunteering substantially and their provision might enable patients to choose from different models.

While the traditional face-to-face volunteering models focus on patients with lower quality of life, supporting them to leave their homes and do more social activities; models of providing volunteering digitally may offer something different, and may require different input from volunteers. These formats of delivery may require different resources (e.g. providing different support to both patients and volunteers or changing the typical training and supervision of volunteers). Furthermore, digital volunteering may also help to recruit new types of volunteers and open up the possibility of volunteering across large distances and even across countries.

Even though digital volunteering may be comparatively easier to arrange than face-to-face encounters, it may bring other challenges and barriers. This could for example encompass patients facing financial costs to use these tools [42], or issues around data confidentiality and data security of the communication.

Although digital volunteering appears appealing to a significant number of patients with psychotic disorders, developmental work and research evaluation is required to design and provide such volunteering support that is also appealing to volunteers and leads to beneficial experiences for both patients and volunteers. The question however if these digital interactions would come as a complement or replacing face-to-face encounters should also be further explored and studied.

The interests that patients expressed of having open-ended relationships with the volunteers, for volunteers to be in contact with the mental health teams, and for organisations to cover expenses, may have implications in practice and policies, and pose challenges to its implementation.

These findings indicate that quality of life is important when considering patients’ likelihood of being interested in face-to-face volunteering programs. Hence, patients with higher interest to seek out a volunteer may also be those who are most in need of it, and are likely to gain more from it. Equally, these findings indicate that younger patients with fewer years of diagnosis were more likely to be interested in digital volunteering, suggesting that offering digital models of volunteering, for example linked with early intervention services, may become more important in the future.

In light of this, future research should investigate the feasibility of face-to-face volunteering targeting the subjective quality of life of people with psychosis, and the feasibility of digital volunteering targeting patients who are younger and at an early stage of their illness.

## Conclusions

The findings indicate that more than half of the patients with psychosis have not heard of volunteer befriending programs. Those with lower quality of life were more likely to express interest in getting face-to-face volunteering input, and patients who were younger and with fewer years of diagnosis were more likely to be interested in getting digital volunteering. This highlights the potential for digital volunteering to increase in the future. The variability of the views from patients with psychosis in this study suggests a need for flexibility and innovation in the design and models offered, with a variety of contact methods, in order to maximise the potential benefits of volunteering.
